# Bulk Surfaces Coated with Triangular Silver Nanoplates: Antibacterial Action Based on Silver Release and Photo-Thermal Effect

**DOI:** 10.3390/nano7010007

**Published:** 2017-01-06

**Authors:** Agnese D’Agostino, Angelo Taglietti, Roberto Desando, Marcella Bini, Maddalena Patrini, Giacomo Dacarro, Lucia Cucca, Piersandro Pallavicini, Pietro Grisoli

**Affiliations:** 1 Department of Chemistry, University of Pavia, Viale Taramelli 12, 27100 Pavia, Italy; agnese.dagostino01@universitadipavia.it (A.D.); desandoroberto.91@hotmail.it (R.D.); marcella.bini@unipv.it (M.B.); giacomo.dacarro@unipv.it (G.D.); lucia.cucca@unipv.it (L.C.); psp@unipv.it (P.P.); 2 Department of Physics, University of Pavia, Via Bassi 6, 27100 Pavia, Italy; maddalena.patrini@unipv.it; 3 Department of Drug Sciences, University of Pavia, Viale Taramelli 14, 27100 Pavia, Italy; pietro.grisoli@unipv.it

**Keywords:** antibacterial surfaces, silver nanoplates, seed-growth synthesis, photothermal effect, hyperthermia

## Abstract

A layer of silver nanoplates, specifically synthesized with the desired localized surface plasmon resonance (LSPR) features, was grafted on amino-functionalized bulk glass surfaces to impart a double antibacterial action: (i) the well-known, long-term antibacterial effect based on the release of Ag^+^; (ii) an “on demand” action which can be switched on by the use of photo-thermal properties of silver nano-objects. Irradiation of these samples with a laser having a wavelength falling into the so called “therapeutic window” of the near infrared region allows the reinforcement, in the timescale of minutes, of the classical antibacterial effect of silver nanoparticles. We demonstrate how using the two actions allows for almost complete elimination of the population of two bacterial strains of representative Gram-positive and Gram-negative bacteria.

## 1. Introduction

Functional nanomaterials are increasingly finding wide success in several research fields devoted to human well-being. Just to name a few recent examples, functional nanomaterials can be used for nanodelivery of anticancer drugs [1] and, more generally, of pharmaceuticals molecules [2], for theranostic purposes in the presence of peculiar plasmonic effects given by properly functionalized noble metal nano-objects [3]. Functional nanomaterials can even to provide new solutions to energy production-related problems, as in the case of catalytic nanoarchitectonics [4]. 

Recent news [5] about the existence of an antibiotic-resistant *Escherichia coli* superbug spread concern throughout the whole biomedical scientific community, but also in media and the general population. Pan-drug-resistant bacteria can be particularly dangerous when infecting materials used in the nosocomial environment: bacterial and biofilm-associated infections involving medical, surgical, and prosthetic surfaces are probably the leading cause of severe nosocomial problems [6,7]. In situations where conventional antibiotics are not able to eliminate bacteria from surfaces, a possible tool is the coating of surfaces with silver nanoparticles (AgNPs), acting as a very efficient antimicrobial agent and representing an alternative to antibiotics [8]. The mechanism of this antibacterial action is still under debate, but it is now accepted that it involves the release of Ag^+^ ions and their subsequent interaction with bacteria, even if one cannot exclude the presence of a short-distance nanomechanical action caused by the direct interaction of AgNPs with the bacterial cell membrane [9]. Usually, AgNPs are much more effective towards Gram-negative (Gram−) bacterial strains than towards Gram-positive (Gram+), due to the fact that Gram+ bacteria present a relatively thick (20–80 nm) and continuous cell wall almost entirely made of peptidoglycan, while Gram− bacteria feature a less thick, less solid, and less resistant peptidoglycan layer (5–10 nm) surrounded by a phospholipidic membrane [10].

While a huge number of papers report the antibacterial effects of spherical AgNPs, until a few years ago, a limited number of studies investigating the antibacterial activity of anisotropic silver nano-objects had appeared. A first fundamental report [11] showed that truncated triangular nanoplates were more efficient than spherical AgNPs in killing *E. coli*, suggesting the existence of a shape-dependent interaction between AgNPs and Gram− bacteria. Recently, the use of anisotropic silver nanoparticles has started to raise a greater interest, driven also by the fact that localized surface plasmon resonance (LSPR) properties of noble metal nano-objects are strongly dependent on size and shape, and that this feature can be exploited to obtain nano-objects having a wide range of colors, a feature which is exploitable in the coating of textile fibers to obtain colorful antibacterial fabrics [12,13]. Recent studies have discussed the antibacterial effects of triangular, truncated triangular and hexagonal plates, and some comparison with spherical AgNPs was attempted [14,15]. In a very recent example, the strong antibacterial activity of anisotropic nanoparticles toward *Pseudomonas aeruginosa* cells was demonstrated when applied to corneal replacements [16]. 

Interestingly, noble metal nanoparticles can be used for antibacterial purposes by also exploiting a different feature: the hyperthermia produced by their photo-thermal effect [17]. Gold nanorods (GNRs) [18] and gold nanostars (GNSs) [19] have been used to this purpose. Even if gold is not an “antibacterial element”, gold nano-objects of anisotropic shape—featuring two (or more) intense LSPR bands—can achieve thermal relaxation after proper irradiation. This produces hyperthermia in their surroundings, which can be used to kill bacteria. When the LSPR band falls in the near-IR range “therapeutic window” (NIR, 750–950 nm) where tissues and blood are semi-transparent, laser irradiation can be used. One can thus imagine applying this strategy to the treatment of possible infections on subcutaneous devices, if they are coated with these kind of nanomaterials. In a recent example, we used a monolayer of GNSs grafted on bulk glass samples [20] to have a model of an active surface. Laser irradiation of this monolayer, exploiting the NIR LSPR absorption of GNSs, yielded an efficient photo-thermal conversion causing hyperthermia, which efficiently kills bacteria cells in *Staphylococcus aureus* biofilms.

The ability to obtain an efficient photo-to-heat conversion is not only limited to gold nano-objects. In two recent and pioneering examples [21,22], the photo-thermal effect given by silver nanoplates was smartly investigated for photo-thermal treatments of cancer cells when irradiated at 800 nm, a wavelength value where a plasmonic band can be easily placed by tuning morphology and dimensions of the objects themselves [23]. Flat silver nano-objects typically show an in-plane dipole plasmon resonance band, whose position can be placed in the NIR region and tuned at a desired value, just by regulating the dimensions and the aspect ratio of the objects [24]. 

Few recent papers have been dedicated to bimetallic nanostructures in which the photo-thermal effects have been studied for augmenting the release of silver, followed by evaluation of this increased release for microbicidal tasks [25,26,27]. Thus, it seems quite odd that no examples are reported in literature on the use of the photo-thermal effects of pure anisotropic silver nanoplates for antimicrobial applications. Only very recently, we have proposed a unique approach, which consisted of growing almost flat silver nanostructures directly on a layer of small AgNP seeds, already grafted on glass. The approach provides for a certain degree of control of the dimensions of the objects obtained, resulting in a quite high surface density of silver (about 6.2 μg/cm^2^) and exploitation of the antibacterial activity achieved by silver release and photo-thermal features [28].

Here, we were interested in a different approach, in order to obtain a more general preparation route. The simple idea is to synthesize anisotropic silver nano-objects with the desired features (shape, dimension, LSPR features, type of coating), and then graft them on a bulk surface. This last step can be easily performed by dip-coating glass samples (properly silanized, introducing a grafting function) in the silver nanoplates colloidal solutions. Once again, we chose glass as an economic material with wide applicability. 

Triangular silver nanoplates can be synthesized in a plethora of ways [23]. In general, anisotropic noble metal nano-objects are prepared by seed-growth methods, and these preparative strategies usually exploit surfactant molecules to produce and direct the anisotropic growth [29,30]. Unfortunately, it can be hard to remove these capping molecules from the nanoparticles surfaces, and this often complicates the chemistry that is needed for further coatings [31]. Also, molecules like surfactants may have a toxic effect on human cells, and this of course is a main issue when nano-objects, in colloidal suspension or grafted on a solid surface, are intended to be used in applications involving human health [32]. Even the presence of polymeric stabilizers, which can improve the growth as well the stability of anisotropic particles [24,33], can represent a problem. Polymeric stabilizers could affect the antibacterial activity [9]—interfering with silver nanoparticle surface oxidation, a step which is needed for subsequent silver ion release [34]—as well their grafting onto surfaces, which can be strongly dependent on the nano-object’s surface charge. On the other side, the citrate anion is not harmful and, when used in seed growth synthesis [23,24,28,29], preferentially binds to (111) facets of growing particles, slowing down the vertical expansion and thus allowing anisotropic growth, producing nanoplates. Moreover, it ensures a negative surface charge, which is crucial for grafting to bulk surfaces functionalized with amines, which are protonated when used in water at neutral or slightly acidic conditions.

**Scheme 1:**
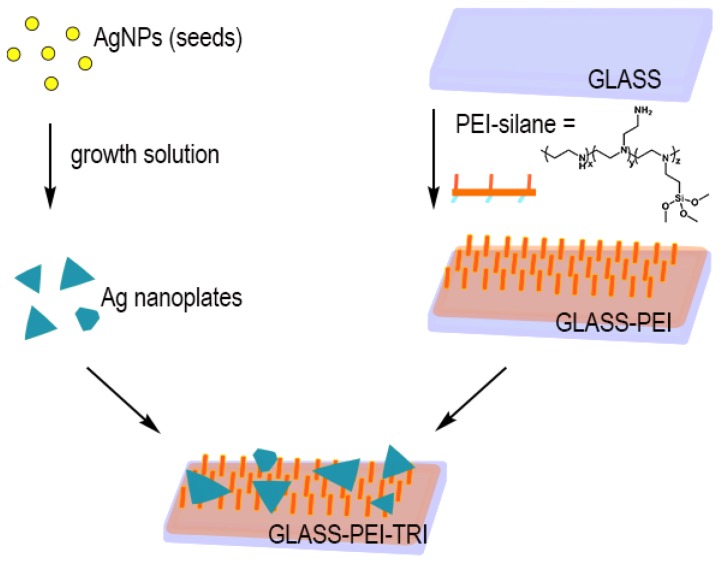
A schematic representation of the synthetic route to polyethylenimine (PEI)-functionalized glass with citrate-capped Ag nanoplates (GLASS-PEI-TRI) samples.

With these considerations in mind, we prepared bulk glass surfaces coated with trimethoxysilylpropyl (polyethylenimine) (PEI-silane, see Scheme 1)—a polymer containing primary, secondary, and tertiary amines—and to these surfaces we grafted opportunely synthesized citrate-coated silver nanoplates to obtain a prototype of a medical device surface [34] carrying a layer of silver nanoplates with the ability to strongly absorb laser radiation in the NIR range. We demonstrate that this coating exerts an antibacterial activity relying on: (i) the well-known microbicidal activity typically obtained with release of silver ions from nano-objects; (ii) the antibacterial action caused by the hyperthermia produced by the photo-thermal effect.

## 2. Results and Discussion

### 2.1. Nanoplates Synthesis

As already stated, we opted for a synthetic route based on a seed-growth method, but avoided any surfactant or polymer in an attempt to produce the nanoplates in absence of any potentially harmful reactant, as well to pursue an economic and “green” preparation and to obtain “safe” silver nano-objects with potential antibacterial action.

Seeds were prepared by modifying a reported method [35], which yielded small spherical nano-objects of about 5 nm diameter. Spectrum of the colloid shows the expected narrow LSPR band centered at about 396 nm. In order to standardize the preparation, seeds were diluted such that stock seed solutions with the LSPR band had an absorbance of 0.5 units.

**Figure 1:**
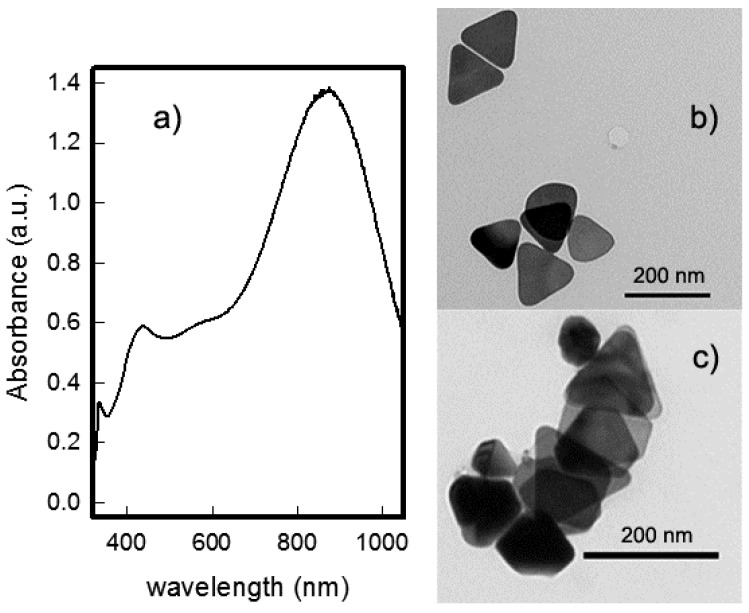
(**a**) UV–vis spectra of colloidal suspension of silver nanoplates obtained with the standard preparation; (**b**,**c**) Transmission electron microscope (TEM) images of silver nanoplates obtained with the standard preparation.

The plates were prepared simply by adding a precise volume of seeds at a proper volume of growth solution, which was composed only by the properly optimized amounts of ascorbic acid, sodium citrate, and silver nitrate. A slow change in suspension color was noted, corresponding to growth of an intense LSPR band, which shifted towards NIR over time while increasing its intensity. The phenomenon was completed in less than 12 h, and no further sensible changes of LSPR features were observed at the time scale of one week, indicating the stability of the obtained colloidal suspension. Completion of nanoplates’ growth is also indicated by the complete disappearance of the UV absorption band of ascorbic acid, centered at 266 nm. The synthesis is quite reproducible when the quantity of seed is strictly controlled, yielding colloidal suspensions where three bands are clearly recognized [33]: as shown in Figure 1a, there is one band at 330 nm (which can be ascribed to the out-of-plane quadrupole resonance for triangular nanoplates), one at about 420–440 nm (the zone where the out-of-plane dipole resonance is usually found), and one that is more intense—the in-plane dipole resonance—at about 845 ± 30 nm (average value based on eight preparations). A spectrum obtained for a representative standard preparation is reported in Figure 1a, while four spectra coming from four repetitions of the identical standard preparation are reported in the Supplementary Material (Figure S4), accounting for an acceptable degree of reproducibility.

It is interesting to note that this last LSPR band can be roughly adjusted by changing the quantity of seed solution added: reducing the seed quantity by 33% results in an LSPR band shifting toward 900 nm, while increasing the added seed quantity to double that of the standard value shifts the final LSPR band close to 800 nm or less. As is obvious, the presence of smaller quantities of seeds produces larger objects with the in-plane dipole resonance moved to higher wavelengths, while the addition of a higher quantity of seeds, when the amount of silver ion precursor and other reactants are kept constant, produces smaller objects with the in-plane dipole band shifted to lower wavelengths.

The complexity of the spectra and the broad absorption band (in the range of 400–650 nm), indicates the presence of several type of different objects.

As shown in Figure 1b,c, transmission electron microscope (TEM) analysis of the suspensions obtained with the standard preparations yielding an in-plane dipolar LSPR placed at 845 ± 30 nm confirms the presence of a majority of triangular plates, with various degrees of snipping of the corners: objects range from almost regular triangles with slightly rounded corners, to a small number of almost hexagonal plates, as can be seen in Figure 1b,c. A certain number of smaller, more rounded objects, as well of objects of other shapes, can be noticed (TEM images in Supplementary Material, Figures S6 and S7). From a few of the objects which were placed vertically in agglomerates observed in TEM images (see Supplementary Material, Figure S6) we estimated an average thickness of about 20 nm. The dimensions of regular triangles obtained with the standard preparation were measured, calculating (as a qualitative parameter for each triangular plate) the three altitude values (defined as the distance between a vertex of a triangle and the opposite side at right angles) and obtaining an average value (coming from four preparations) of 120 ± 25 nm. A histogram representing the distribution of the average altitude value for 43 regular triangles obtained from TEM images coming from the standard preparation reported in Figure 1a is shown as an example in Figure S8 in the Supplementary Material.

As already deduced from LSPR spectra, changing the quantity of seed solution added produces a variation in the dimensions of nanoplates obtained, without altering the mean morphology of the objects. Reducing the seed quantity (33% compared to the quantity used in the standard preparation) results in an enlargement of objects (see TEM images reported in Supplementary Material, Figure S9), with regular triangles’ average altitudes reaching up to 200 nm. Increase of the seed quantity (to double that of the quantity used in the standard preparation) produces smaller objects (with average altitudes of regular triangles reduced to about 80–100 nm), as can be seen in the TEM images reported in Figure S10 in Supplementary Material. In both cases, changes in the morphologies of the obtained objects in the colloids were not observed. 

We must stress that it was not the aim of this work to study the effects of the fine variations of synthetic parameters (such as the quantity of seeds added) on nanoplates’ size and morphology and on the distribution of object shapes. We simply performed a rapid screening, and we were satisfied to find the synthetic conditions needed to produce colloidal suspensions with an intense in-plane dipolar LSPR at the desired wavelength value. The preliminary synthetic investigation shown here should be expanded and described more accurately, and this topic is actually the subject of a more detailed investigation in our laboratory.

Measurements of the ζ-potential of the colloid gave a value of −40.6 ± 4.5 mV, suggesting quantitative coating of the objects with citrate and explaining the high stability of the suspensions, as citrate provides enough surface charge to stabilize particles against aggregation. Stability was also demonstrated by the fact that the colloidal suspension of nanoplates can be centrifuged at 5000 rpm for 15 min and then resuspended in water without any visible change in LSPR spectra. The role of citrate as a stabilizing agent with a shape-directing action is well known: citrate anions preferentially bind to (111) facets of the crystals, which grow after the seed addition to the growth solution, slowing down the vertical expansion and thus obtaining anisotropic growth of the nanoplates. This was also confirmed by X-ray diffraction (XRD) patterns (see Supplementary Material, Figure S15) obtained from a sample of the colloidal suspension. The suspension was centrifuged, concentrated, deposited on blank glass, and dried, obtaining a spot of solid powder of nanoplates. Only the (111) reflection of a face-centered cubic (FCC) structure is observed, indicating that the basal lattice plane of nano-objects is (111), as expected from the growth-directing action of citrate [23].

It is important to stress the fact that a rough but (for our purposes) useful control on the plates’ morphology and dimensions was obtained using only ascorbate and citrate, and by controlling the quantity of seeds. In absence of surfactant or polymeric stabilizers, we obtained a colloid with an LSPR spectrum that fits our need for intense NIR absorption in the so-called “therapeutic window”. In addition, citrate ensures a high negative charge on nanoplates, which is crucial for colloidal stability and for interaction with protonated amines (which will be present on bulk glass surfaces as a grafting layer), while avoiding the use of harmful coating agents. Moreover, AgNPs prepared in presence of citrate usually show very efficient antibacterial action [9,10,34,35,36], a fact which is not ensured in presence of coating agents that could interfere with the release of silver ions.

### 2.2. Grafting of Nanoplates: Preparation and Characterization of Polyethylenimine (PEI)-Functionalized Glass with Citrate-Capped Ag Nanoplates (GLASS-PEI-TRI) Samples

The well-known antibacterial action of AgNPs can be easily brought to macroscopic scale. Recently, we performed this task in several ways; for example, by creating a firmly grafted AgNPs monolayer on glass (used as a model surface), exploiting the Ag–S bond after obtaining a –SH layer on bulk glass by means of the formation of a self-assembled monolayer (SAM) of mercaptopropyltriethoxy silane (MPTS) [34]. We obtained similar results using electrostatic interactions between ammonium groups and negative charges present on AgNPs surfaces: a monolayer of citrate-capped AgNPs can be obtained through preliminary silanization of glass surfaces with 3-aminopropyltriethoxysilane (APTES), [36]. The same can be done preparing a layer of PEI-silane on glass as an adhesive layer [37]. In all these cases, we were able to obtain excellent stability of the nano-objects’ layer in aqueous media, with prolonged release of silver ions, controlled local concentration of Ag^+^ without any detachment of AgNPs, and a strong antibacterial action with limited risks towards human health. Here, we followed the same approach, and prepared a PEI-terminated surface ready to graft silver nanoplates, which are negatively charged (pH of colloidal suspension is around 6 in these conditions, with a distinctly negative ζ-potential as reported in the previous paragraph), because of citrate coating. PEI-silane (see Scheme 1) is a polymer composed of an average of 3.6 propyltrimethoxysilane groups and 56 ethylenimine monomers (i.e., 56 amine moieties (including primary, secondary and tertiary amines). Each polymer molecule has multiple alkoxysilane attaching points. In the standard procedure, 21 × 26 mm glass slides were cleaned and activated with piranha solution, producing an –OH rich, highly hydrophilic surface, having a contact angle < 10° and ready to react quickly with PEI-silane in ethanolic solution, yielding PEI-functionalized surfaces (GLASS-PEI). As a characterization step, static contact angle (49° ± 4°) was measured after functionalization, in good agreement with our previous results [28,37].

**Figure 2:**
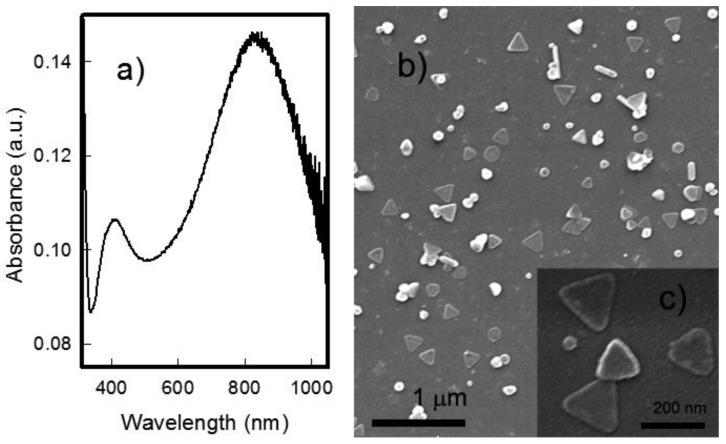
(**a**) UV–vis spectra of a GLASS-PEI-TRI sample; (**b**) Scanning electron microscope (SEM) image of a GLASS-PEI-TRI sample; (**c**) detail of a high magnification SEM image showing the presence of triangular nanoplates on the GLASS-PEI-TRI samples.

Grafting of nanoplates was obtained by dipping the GLASS-PEI slides for 14 h in aqueous colloidal suspensions of citrate-capped Ag nanoplates. The typical UV–vis–NIR spectra of glass samples obtained after this treatment (GLASS-PEI-TRI), reported in Figure 2a, clearly shows the grafting of the nanoplates. The out-of-plane dipole band in the zone around 400–450 nm can be recognized. The in-plane dipolar band is centered in the 820 ± 30 nm range, with a 20–30 nm blue-shift with respect to the starting colloidal suspension. This shift is essentially due to the decrease of the local refractive index (*n*) on passing from water (*n*_water_ = 1.3339) to glass and air at the interface (*n*_air_ = 1.0003) [34]. The out-of-plane quadrupole resonance band observed at 330 nm cannot be found, probably because it is too weak and covered by the absorbance of glass itself in the UV region. Once again, broadening of the LSPR bands can be explained by the presence of objects with different morphologies and dimensions. Visually, the samples show a very light grey-blue color.

Figure 2 also shows a couple of scanning electron microscope (SEM) images for GLASS-PEI-TRI samples at different magnifications. As can be seen in the image reported in Figure 2b, a homogeneous distribution of all the objects already observed in the TEM images was obtained, with the presence of some agglomerations of objects that still retain dimension and morphology. This can be more precisely observed in Figure 2c, where some regular triangles are shown. The homogeneous and well-spaced distribution on the surface allowed us to calculate the composite percent of different objects obtained in the seed-growth synthesis (already noticed in TEM images) and grafted on glass with this last step. Counting the objects found in large area SEM images gave the following constant composition: 49% of regular triangles, 19% of triangles with various degrees of snipping (up to hexagonal shape), 12% of rod-shaped objects, and 20% of more rounded, discoidal objects. Size measurement of several regular triangles gave a value of 125 ± 25 nm for their altitude, in good agreement with the values derived from TEM.

The preparation of bulk glass samples coated with silver nanoplates is quite reproducible: in Figure S11, spectra of four samples coming from four identical (but obtained in four different reaction vessels) preparations obtained from the same colloidal suspension are reported, while Figure S12 reports the spectra of seven samples obtained in a single preparation (i.e., in the same reaction vessel). GLASS-PEI-TRI slides are very stable in air (i.e., their spectra did not change significantly for at least a 2-month period). 

XRD patterns of GLASS-PEI-TRI samples showed once again only the presence of a (111) peak. In this case, a certain broadening is observed when the peak is compared to the one found in the case of the nanoplates powder (see Figure S15 in Supplementary Material). This broadening can be explained on the basis of the relatively low surface concentration of plates in the GLASS-PEI-TRI samples.

We also carried out the characterization of the GLASS-PEI-TRI with quantitative oxidation (using controlled amounts of strong acid) of the silver nanoparticles that had been grafted on the samples, and analyzed the obtained solution by means of inductively coupled plasma (ICP). Data from eight different samples, coming from eight different preparations, gave an average of 1.87 (0.30) × 10^−6^ g/cm^2^ of silver. This quantity is higher than those found for small spherical AgNPs monolayer samples [34,36,37], but sensibly lower than the described case in which a seed-growth synthesis of flat silver objects is performed directly on glass [28].

A feature of primary interest for the applications we have in mind is the ability to release a controlled quantity of silver without detachment of nano-objects in the environment, when the sample is immersed in an aqueous environment. We investigated this aspect by placing freshly prepared GLASS-PEI-TRI samples in ultrapure water for 48 h, and then measuring their UV–vis–NIR spectra and examining their SEM images (see Figures S13 and S14 in Supplementary Material). We also measured the UV–vis–NIR spectra of obtained solutions, which were subsequently analyzed with ICP. Data from the solutions obtained after 48 h immersion in ultrapure water of GLASS-PEI-TRI samples showed the presence of 0.075 (0.030) × 10^−6^ g/cm^2^ of silver. UV–vis–NIR spectra on the same solutions did not show any LSPR absorbance, thus ruling out the presence of plasmonic nano-objects and detachment of objects from the surfaces. Consequently, all the silver found in solution is ionic, and not belonging to detached metallic nano-objects. This was confirmed from SEM images on the samples extracted from the solutions after the immersion, which showed the same homogeneous distribution of the objects that was observed in the freshly prepared samples, with objects conserving their average number, morphology, and size; this can be observed by comparing Figure 2 and Figure S14. Moreover, the UV–vis–NIR spectra of the same GLASS-PEI-TRI samples are almost identical to the spectra registered before the immersion. Even if the situation in more complex media is sensibly different, these experiments indicate that: (i) the objects are stably grafted on the surfaces; (ii) there is a limited release of silver ions from these objects, which (compared to the total quantity of silver found on the samples) means that only about 4% of total grafted silver is released after 48 h of immersion in water; and (iii) this release neither significantly changes the shape and dimensions of the objects, nor their consequent LSPR features.

**Figure 3:**
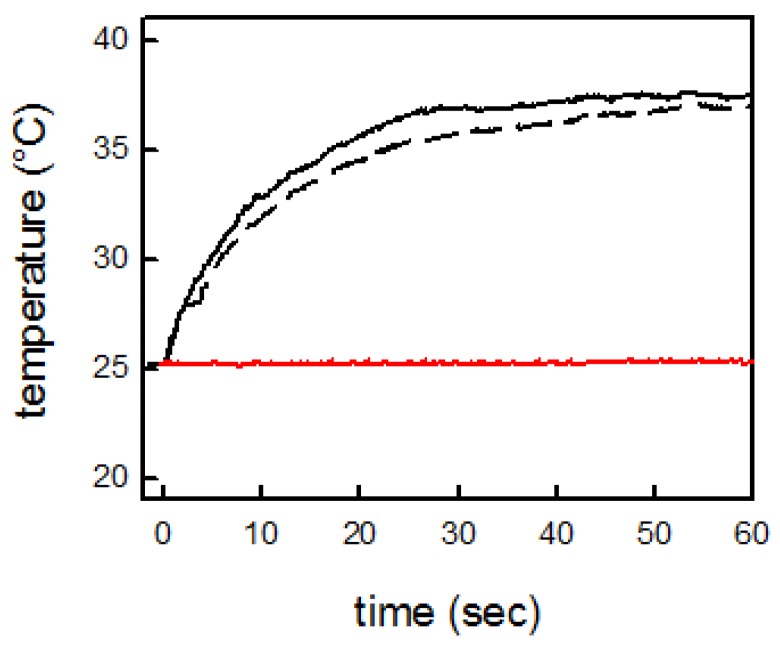
Temperature increase as a function of irradiation time, measured under NIR laser irradiation (808 nm, 0.26 W/cm^2^) for a freshly prepared GLASS-PEI-TRI sample (black solid line) and for a GLASS-PEI-TRI sample after an immersion of 48 h in water (black dashed line). The red solid line represents the thermogram obtained for an uncoated glass in the same conditions.

At this stage, we wanted to check the photo-thermal features of the grafted nano-objects (i.e., their ability to produce localized hyperthermia when properly irradiated). To do this, GLASS-PEI-TRI samples showing an LSPR maximum around 830 nm were irradiated with a laser source at an 808 nm with a power of 200 mW, using a spot of 1.0 cm in diameter, corresponding to an irradiance of 0.26 W/cm^2^. Using a thermocamera, the temperature in correspondence to the irradiated spot was measured and plotted against time of irradiation. As can be clearly seen in Figure 3, irradiation causes a temperature increase of about 11–12 °C in a few seconds. The same behavior is observed when the measure is repeated on a sample after 48 h of immersion in water, demonstrating once again that a prolonged immersion in water does not produce any change in plasmonic features of the nanoplates layer. As is predictable, irradiation of uncoated glass samples in the same conditions does not produce any measurable hyperthermia.

### 2.3. Antibacterial Features of GLASS-PEI-TRI Samples

As a first step to evaluate the antibacterial features, we investigated the microbicidal effect (ME) of GLASS-PEI-TRI samples against *E. coli* and *S. aureus*, which are representative strains of Gram− and Gram+ bacteria, respectively. We have recently introduced a procedure which allows the evaluation of the ME of a properly coated surface in contact with a thin liquid film containing bacteria, using the following formula:

ME = log *N*_C_ − log *N*_E_, (1)

where *N*_C_ is the number of CFU/mL developed on the unmodified control glass, and *N*_E_ is the number of CFU/mL counted after exposure to modified glass (CFU = colony forming unit).

We chose three contact times (15 min, 5 h, and 24 h) and evaluated the ME after these intervals, which are reported in Table 1. The longer contact time was set to 24 h, as biofilm colonies usually develop on biomaterials in a 12–18 h period from the bacterial contamination: thus, an antibacterial activity lasting for 24 h is an optimal feature to impart the ability to prevent biofilm growth to biomedical surfaces [36]. Results show that GLASS-PEI-TRI glass slides exert a significant bactericidal effect that increases with the contact time. For *E. coli*, a contact time of 5 h is enough to eliminate at least 99.9% of bacteria cells. After 24 h, more than 99% of *S. aureus* are killed too. It is important to notice that tests with contact times longer than 24 h suffer from a main limitation: in the conditions of the experiment, in the timescale of days, the bacteria in the control samples also tend to die, thus reducing the number of colony forming units and giving poorly reproducible and reliable values of ME. Anyway, preliminary tests performed with a 48 h contact time showed that ME does not increase when the contact time is prolonged for more than 24 h. The effect on *S. aureus* seems a little less intense with 5 h of contact, while no microbicidal effect towards either strain is evident within the short (15 min) contact time. This is consistent with precedent studies on AgNP-coated antibacterial surfaces: as we have reported for similar systems [34,36,37], one should expect a very limited release of silver ions for short (less than 1 h) contact times, and this explains the low ME observed in these timescales. Mechanisms by which AgNPs exert their antibacterial effect is still debated; however, it was recently shown [38] that silver nano-objects are devoid of antibacterial action when used under anaerobic conditions, which exclude oxidation to silver (I), thus preventing its release. This is consistent with our proposal [34] of the formation of an oxidized silver layer on the surface of citrate-coated grafted nano-objects, reaching a steady-state in which Ag^+^ ions are slowly released, continuously being replaced by Ag oxidized from the bulk of the nano-objects. Remarkably, in the tests proposed by CEN (European Committee for Standardization) in EN 13697, the microbicidal activity of a disinfectant is considered acceptable when the decimal-log reduction rate (which is strictly related to our ME) is at least equal to 4 after 5 min of contact. In our case the ME of the modified glass is very close to 4 after 24 h of contact with *E. coli*, showing a long-term action that seems well suited for building antibacterial surfaces for medical devices.

This antibacterial effect, anyway, is not a surprise: it was largely expected and substantially confirms the behavior obtained with spherical nanoparticles. We were much more interested in the new features that are introduced by the anisotropic shape on nanoplates (i.e., the possibility to efficiently absorb NIR laser radiation, to dissipate it as heat, and to exploit this hyperthermia to kill bacteria cells). In literature, no precise information is present concerning the temperature values needed to eliminate bacteria with hyperthermia caused by a photo-thermal effect. It is reported that the heating of *E. coli* cells at temperatures of 52 °C for a time longer than 5 min causes the destruction of the permeability barrier of bacteria cells [39]. It is also reported that laser irradiation of a cluster of gold nanorods for 5 min allows a temperature of about 60 °C to be reached, thus destroying most bacterial cells in a biofilm of *E. coli* [18]. Recently it was described how NIR laser irradiation of a monolayer of GNSs on a glass bulk surface for 30 min causes a few degrees hyperthermia, which is enough to almost completely destroy a bacteria colony in a *S. aureus* biofilm layer [20]. In any case, we must stress the fact that “macroscopic” effects, especially when described by thermograms registered with a thermocamera, only show temperature changes of relatively large areas (in the range of 1 cm^2^) of bulk glass samples, changes which are, of course, the function of several experimental parameters [19]. So, information on the temperatures that are reached in the close surroundings of the nano-objects’ surfaces are completely missed, even if one can reasonably state that these temperatures are considerably higher than those measured by thermograms. From the reported literature data, anyway, it is possible to speculate that also a few degrees hyperthermia measured on a bulk surface could be useful to produce a strong effect on bacteria health [28].

Thus, we moved toward the investigation of the antibacterial action obtained by laser-induced photo-thermal effects causing localized hyperthermia. GLASS-PEI-TRI samples—reduced to appropriate dimensions in order to be completely covered by the laser spot—were irradiated for 15 min with an 808 nm laser source, while in contact with the described bacteria suspensions, using an irradiance of 0.26 W/cm^2^. In this case, the “thermal microbicidal effect”, ME_T_, was calculated with the following formula:

ME_T_ = log *N*_C_ − log *N*_T_, (2)

where *N*_C_ is the number of CFU/mL developed after the 15 min contact with GLASS-PEI-TRI samples in absence of irradiation, taken as control, and *N*_T_ is the number of CFU/mL counted after 15 min of contact with GLASS-PEI-TRI samples in presence of NIR laser irradiation. It is important to stress that, in these conditions, ME_T_ must be ascribed only to the photo-thermal effect, as any possible influence from the (limited) silver release occurring in absence of irradiation is already considered in the control count. Moreover, we have the evidence that no silver-related ME is observed during the first 15 min of contact, as already reported in Table 1. It is also important to stress that when the experiment is repeated using nonfunctionalized glass samples, the ME_T_ is absent (<0.3). This clearly indicates that laser irradiation alone, in absence of plasmonic objects exerting photo-thermal features, does not cause any harm to bacteria. The same set of experiments were replicated on a set of samples after their immersion in water for 24 h, showing the same ME_T_ values, thus confirming stability of the sample features after long immersions times in water.

The ME_T_ test results show that the laser treatment kills more than 99.7% of *E. coli* and about 97% of *S. aureus* cells in 15 min of irradiation at 0.26 W/cm^2^, a value which is below the maximum allowed for exposure of the skin [40], and using a wavelength which is suitable for in vivo use. As reported in Table 2, in the case of ME_T_, we observe a higher activity against *E. coli* than against *S. aureus*, which was somewhat already observed in the values of ME that were due only to silver release. This may be due to the fact that Gram-positive bacteria present a relatively thick (20–80 nm) and continuous cell wall consisting mainly of peptidoglycan, while Gram-negative bacteria feature a thinner peptidoglycan layer (5–10 nm) surrounded by an outer phospholipidic membrane, which is expected to be less solid and less resistant [9,10,28]. In this case, hyperthermia caused by the photo-thermal effect seems particularly efficient in killing Gram-negative bacteria. As immersion in water for 24 h does not change the shape, dimensions, LSPR features, or the photo-thermal behavior of objects, it obviously has no effect on hyperthermia and subsequently induced antibacterial activity, which is conserved. This behavior allows one to imagine the use of the laser switched, hyperthermia-related, thermal ME after the surface has already exerted its silver release-related microbicidal action (described by ME values reported in Table 1), in all the cases in which an “extra” action is needed.

## 3. Materials and Methods

### 3.1. Materials

Silver nitrate (*>*99.8%), sodium borohydride (*>*99.0%), sodium citrate (TSC) (*>*99.0%), and ascorbic acid ≥99% were purchased from Sigma-Aldrich (St. Louis, MO, USA)). Trimethoxysilylpropyl (polyethylenimine) (50% in isopropanol) was purchased from Gelest Inc. (Morrisville, PA, USA). Reagents were used as received. Solvents were purchased from Sigma-Aldrich (St. Louis, MO, USA)) and used as supplied. Microscopy cover glass slides (21 × 26 mm) were purchased from DelChimica (Milano, Italy). Water was deionized and then bi-distilled.

### 3.2. Seeds Preparation

Seeds, spherical silver nanoparticles, were prepared according to a reported method [35]. To 20.0 mL of distilled water, the following solutions were added in sequence under vigorous stirring: 0.100 mL of a solution of AgNO_3_ 0.018 M, 0.100 mL of a solution of TSC 0.017 M, and 0.600 mL of a solution of NaBH_4_ 0.010 M. After the last addition, stirring was immediately stopped in order to avoid coagulation. The colloidal suspensions were stored in the preparation flask and maintained in the dark. Then, this colloidal suspension of seeds was diluted with distilled water to reach an absorbance value of 0.5 units to obtain a stock solution, which was used for preparation of silver nanoplates.

### 3.3. Synthesis of Silver Nanoplates

Silver nanoplates were synthesized by a seed-mediated growth method. Firstly, growth solution was prepared in a beaker under magnetic stirring, adding 20.0 mL of a solution of TSC 8.5 × 10^−3^ M, 12 mL of a solution of AgNO_3_ 5 × 10^−4^ M, and 0.300 mL of ascorbic acid 1 × 10^−2^ M. Secondly, 0.300 mL of the stock solution of seeds was added and magnetic stirring was instantly stopped. A changing color of solution could be observed from pale yellow to dark pink, and finally to a blue/grey color, after approximately 12 h. Silver nanoplates colloids were stored at 25 °C and used without further treatments.

### 3.4. Preparation of PEI-Silane Self-Assembled Monolayers on Glass Surface (SURF-PEI Glass)

The samples were prepared according a reported method [28]. Briefly: glass substrates were cleaned for 30 min in freshly prepared piranha solution (3:1 *v*/*v* H_2_SO_4_:H_2_O_2_ (30%) (Caution! Piranha solution is a strong oxidizing agent and should be handled with care). Glass substrates were then washed three times with ultrapure water in a sonic bath and oven-dried. The slides were then immersed for 6 min in a 4% (*v*/*v*) solution of PEI-silane in ethanol at room temperature. In a typical preparation, eight glass slides were prepared at the same time (i.e., reacting in the same silane solution inside an eight-place holder (a microscope glass slides staining jar)). After this, the slides were washed two times with ethanol and one time with ultrapure water in a sonic bath and blow-dried with nitrogen.

### 3.5. Preparation of GLASS-PEI-TRI

The silver nanoplates were grafted on SURF-PEI modified glass slides. In a typical preparation, eight glass slides were covered with a colloidal suspension of silver nanoplates inside an eight-place holder (a microscope glass slide staining jar) for 14 h at 25 °C. Finally, functionalized glass slides were rinsed three times with distilled water and gently dried under nitrogen flux.

### 3.6. Antibacterial Activity Tests

The antibacterial activity of functionalized cover glasses was investigated against *Staphylococcus aureus* ATCC 6538 (Gram+) and *Escherichia coli* ATCC 10356 (Gram−). The microorganisms were grown overnight in Tryptone Soya Broth (Oxoid; Basingstoke, Hampshire, UK) at 37 **°**C. Washed cells were resuspended in Dulbecco’s phosphate-buffered saline (PBS) and optical density (OD) was adjusted to 0.2 at 650 nm wavelength, corresponding approximately to 1 × 10^8^ CFU/mL. Bacterial suspension (10 µL) was deposited onto a standard glass slide (76 × 26 mm), then the microbial suspension was covered with a functionalized cover glass slide (21 × 26 mm), forming a thin film between the slides that facilitates direct contact of the microorganisms with the active surface. The two assembled glasses were introduced in a Falcon test tube (50 mL) containing 1 mL of PBS to maintain a damp environment. In this test, microbes are incubated in non-nutritive suspensions that do not give the microorganisms the potential to grow during the test. For each bacterial strain, three equivalent modified glass slides were prepared; the slides were maintained in contact with the liquid films containing bacteria at room temperature for 15 min, 5 h, and 24 h, respectively; for each time of contact, an unmodified glass slide was treated in the same way as control sample. After the specified times of contact had elapsed, 9 mL of PBS was introduced in each Falcon test tube under gentle shaking to detach the assembled glass slides. Bacterial suspensions were then grown in Tryptone Soya Agar (Oxoid; Basingstoke, Hampshire, UK) to count viable cells. The decimal-log reduction rate (i.e., the microbicidal effect (ME)) was calculated using the formula:

ME = log *N*_C_ − log *N*_E_ (3)

(*N*_C_ being the number of CFU/mL developed on the unmodified control glasses, and *N*_E_ being the number of CFU/mL counted after exposure to modified glasses). The results expressed as ME represent the average of three equivalent determinations. This method can be considered as a version of the Japanese Industrial Standard JIS Z 2811 developed to measure the antibacterial activity of plastic surfaces.

### 3.7. Thermal Microbicidal Tests

Antibacterial activity due to the photo-thermal effect of was investigated against *Staphylococcus aureus* ATCC 6538 (Gram+) and *Escherichia coli* ATCC 10356 (Gram−). One functionalized slide was cut in four sections of 10 × 10 mm in order to be completely irradiated by laser. A volume of 0.020 mL of bacterial suspension was deposited on two sections. For each pair of functionalized glass slides, one was irradiated for 15 min, whereas the other was not irradiated. After this time, the glass section covered with bacterial suspensions was suspended in 1 mL of sterile water, then it was taken away and water was used to make three dilutions in 3 different tubes, in which 5 mL of sterile water was contained: 1:100, 1:10,000, 1:100,000. From each tube, 1 mL of suspension was taken and then grown in Tryptone Soya Agar (Oxoid; Basingstoke, Hampshire, UK) to count viable cells. The decimal-log reduction rate (i.e., the “thermal microbicidal effect”) ME_T_ was calculated with the following formula

ME_T_ = log *N*_C_ − log *N*_T_ (4)

where *N*_C_ is the number of CFU/mL developed in contact with a nonirradiated modified control glass sample, and *N*_T_ the number of CFU/mL counted after exposure to modified glass samples and irradiation. Each experiment was repeated three times.

### 3.8. Instrumentation and Instrumental Methods

Absorbance spectra of colloidal suspensions were taken with a Varian Cary 100 spectrophotometer (Agilent, Santa Clara, CA, USA) in the 200–1100 nm range, and spectra of functionalized glass slides were obtained placing the slides on the Varian Cary 100 spectrophotometer equipped with a dedicated Varian solid sample holder.

TEM images were obtained on colloidal solutions of silver nanoplates diluted five times with distilled water, deposited on Nickel grids (300 mesh), covered with a Parlodion membrane, and observed with a Jeol JEM-1200 EX II instrument (Jeol Ltd., Tokyo, Japan).

Zeta potential was measured with a Zetasizer Nano ZS90 Malvern instrument Malvern Instruments Ltd., Malvern, UK), equipped with dedicated cuvettes. Measurements were repeated three times using 1 mL of colloidal suspension for each one.

SEM images were taken from Tescan Mira XMU variable pressure field emission gun scanning electron microscope—FEG SEM (Tescan USA Inc., Warrendale, PA, USA). Slides were mounted onto aluminum stubs using double-sided carbon adhesive tape and were then made electrically conductive by coating in vacuum with a thin layer of Pt/Pd (2.5 nm). Observations were made in backscattered electrons mode (BSE) at 30 kV and with InBeam secondary electron detector for higher spatial resolution.

Thermograms (temperature vs. time) were recorded on SURF-PEI-TRI samples by means of a laser (multimode AlGaAs laser diode, L808P200, Thorlabs GmbH (Thorlabs GmbH Dachau/Munich, Germany), emitting light at the wavelength of about 808 nm, power of radiation is 200 mW, irradiance is 0.26 W/cm^2^). Temperature was recorded by means of a FLIR E40 thermocamera and FLIR Tools + software (FLIR systems, Wilsonville, OR, USA).

The total Ag content of GLASS-PEI-TRI was determined by quantitatively oxidizing the silver plates grafted on a single slide by dipping it in 3 mL ultrapure concentrated HNO_3_ diluted 1:5 with water (13% *w*/*v* as final concentration) in a beaker, and keeping it overnight at RT on a Heidolph Promax 1020 (Heidolph Instruments GmbH & Co. KG, Schwabach, Germany) reciprocating platform shaker. The Ag content in solution was then determined by inductively coupled plasma optical emission spectroscopy (ICP-OES) with an ICP-OES OPTIMA 3000 PerkinElmer instrument (Perkin Elmer Inc., Waltham, MA, USA). The measure was repeated on eight glass samples coming from four different preparations.

Release of Ag^+^ in water was measured on a set four 4 functionalized slides (21 × 26 mm coated on both sides, total coated surface 10.92 cm^2^) prepared as described above. Each slide was then immersed in 3 mL of ultrapure water. Slides were taken off the water after 48 h. A UV–vis spectra was measured on the water solution, and then the content of Ag^+^ was determined by ICP-OES. Measurements were repeated three times, and mean values are given. ICP data were collected with an ICP-OES OPTIMA 3000 PerkinElmer instrument (Perkin Elmer Inc., Waltham, MA, USA). UV–vis spectra were collected on the glass samples after the immersion. One glass sample was then analyzed by SEM (Tescan USA Inc., Warrendale, PA USA). 

To prepare a sample of nanoplates synthesized with standard preparation for XRD measurement, 10 mL of colloidal suspension was centrifuged for 20 min at 5000 rpm and resuspended in 3 mL of bi-distilled water. This volume was centrifuged again, resuspended in a drop of water, and finally dropped on a blank glass slide and dried for one night. XRD of GLASS-PEI-TRI samples were measured directly on a functionalized slide obtained as described in Section 3.5. XRD measurements were performed by using a Bruker D5005 diffractometer (Bruker, Billerica, MA, USA) with the Cu Kα radiation, graphite monochromator and scintillation detector. The patterns were collected with a step size of 0.03° and counting time of 2 s per step in the angular range 20°–65°.

## 4. Conclusions

The GLASS-PEI-TRI samples exert an antibacterial action, which seems particularly active for Gram− bacteria like *E. coli*, exploiting two separate mechanisms. One is the long-term action given by the “classical” silver ion release, which can be used to prevent the formation and adhesion of colonies over a long period of time, while the other is the laser-switchable photo-thermal action, which can be activated based on need (for example, in the case of infections not sufficiently prevented by the first mechanism) and obtained using the peculiar spectral features of the objects grafted on the surface, objects which can be tailored in order to have their LSPR spectra matching with the wavelength of the desired laser source.

In our case, the laser-switched antibacterial activity is obtained by irradiation of bulk surfaces in the NIR (which could be used in vivo) using a radiation intensity which does not damage the tissues. LSPR absorption was placed close to the laser wavelength at 808 nm, using a simple synthesis which avoids reactants that can be dangerous to human health. The typical antibacterial features usually observed in surfaces coated with silver nano-objects—which are due essentially to the controlled release of Ag^+^ ions, and which is ensured by the verified stability of the grafted nano-objects layers—are conserved. As glasslike SiO_2_ films can be easily deposited on a large spectrum of materials by gelification of a siloxane sol, the procedures herein described—involving a first silanization step of a glass sample—can also be applied to coat a wide range of materials. Thus, these results could be useful for applications to several types of materials of medical use (prosthetic and subcutaneous devices, surgical sutures, wound dressings, to name a few examples). All these kinds of bulk materials surely need long-term antibacterial surface protection, here ensured by the ability of silver nano-objects to release small and controlled amounts of silver ions for long contact times (some days), but will also profit by a switchable, very quick (a few minutes) laser treatment to ensure an “extra” elimination of bacteria cells, when needed.

## Figures and Tables

**Table 1:** Microbicidal effect (ME) values ^1^ for GLASS-PEI-TRI samples.

Bacterial Strain	15 min	5 h	24 h
***E. coli***	<0.3	3.2 (0.3)	3.6 (0.3)
***S. aureus***	<0.3	0.9 (0.3)	2.5 (0.3)

^1^ Values are obtained as the average of three measurements. ME = log *N*_C_ − log *N*_E_.

**Table 2:** Thermal ME (ME_T_) values ^1^ for GLASS-PEI-TRI samples.

Bacterial Strain	15 min	15 min (after 24 h in Water)
***E. coli***	2.5 (0.3)	2.4 (0.3)
***S. aureus***	1.5 (0.3)	1.5 (0.3)

^1^ Values are obtained as the average of three measurements. ME_T_ = log *N*_C_ − log *N*_T_.

## Supplementary Materials

The following are available online at http://www.mdpi.com/2079-4991/7/1/7/s1, Figure S1: TEM image of seeds preparation, Figure S2: UV–vis spectra of different stock solutions of seeds, Figure S3: UV–vis spectra showing the kinetic of evolution with time of a typical seed-growth preparation of silver nanoplates, Figure S4: UV–vis spectra of different colloidal suspensions of silver nanoplates obtained with the standard preparation, Figure S5–S7: TEM images of objects obtained for standard nanoplates preparations, Figure S8: Histogram representing the distribution of the average altitude values measured for 43 regular triangles obtained from a standard preparation. Figure S9: TEM images of nanoplates obtained decreasing of one third the seed quantity (in respect to the standard preparation). Figure S10: TEM images of nanoplates obtained doubling the seed quantity (in respect to the standard preparation), Figure S11: UV–vis spectra of four GLASS-PEI-TRI samples obtained in four different reaction vessels, coming from four identical preparations. Figure S12: UV–vis spectra of seven GLASS-PEI-TRI samples obtained in the same reaction vessel during a single preparation. Figure S13: UV–vis spectra of the same GLASS-PEI-TRI sample before (solid line) and after (dashed line) immersion in water for 48 h, Figure S14: SEM images of sample of GLASS-PEI-TRI after 48 h of immersion in water: (a) large area image; (b–d) images of triangular objects, which retains morphology and dimension observed in the fresh samples, Figure S15: XRD data.
