# Supplementary material for: Bulk Surfaces Coated with Triangular Silver Nanoplates: Antibacterial Action Based on Silver Release and Photo-Thermal Effect
Source: Nanomaterials (Basel). 2017 Jan 6;7(1):7. doi: 10.3390/nano7010007 (PMC5295197; doi:10.3390/nano7010007)
Supplement: Supplementary file 1 [file nanomaterials-07-00007-s001.pdf]

Supplementary Materials: Bulk Surfaces Coated with Triangular Silver Nanoplates: Antibacterial Action Based on Silver Release and Photo-Thermal Effect

Agnese D'Agostino, Angelo Taglietti, Roberto Desando, Marcella Bini, Maddalena Patrini, Giacomo Dacarro, Lucia Cucca, Piersandro Pallavicini and Pietro Grisoli

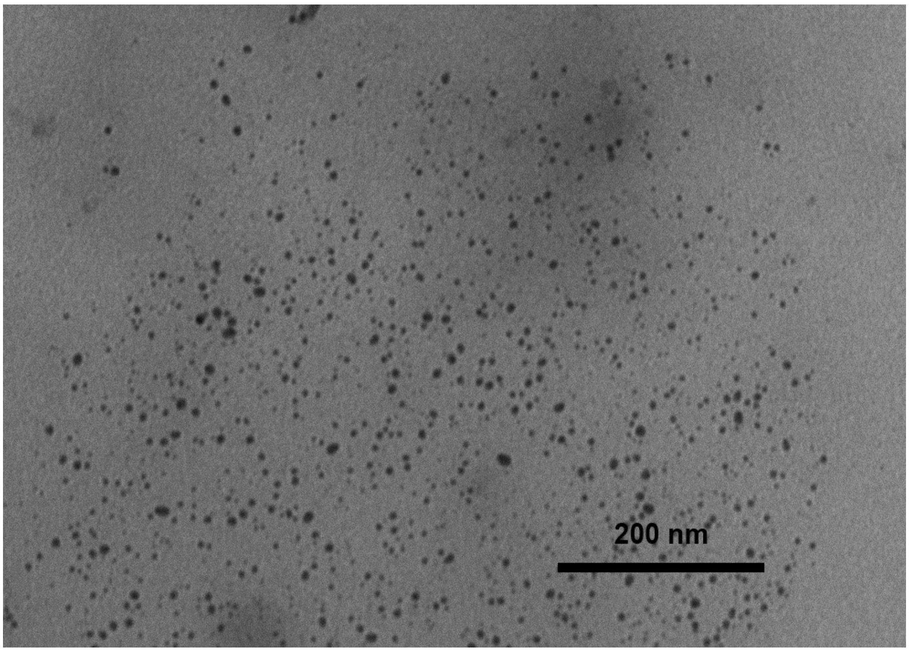

Figure S1. TEM image of seeds preparation, showing the presence of spherical nanoparticles with a mean diameter of 5.4 ± 2.6 nm.

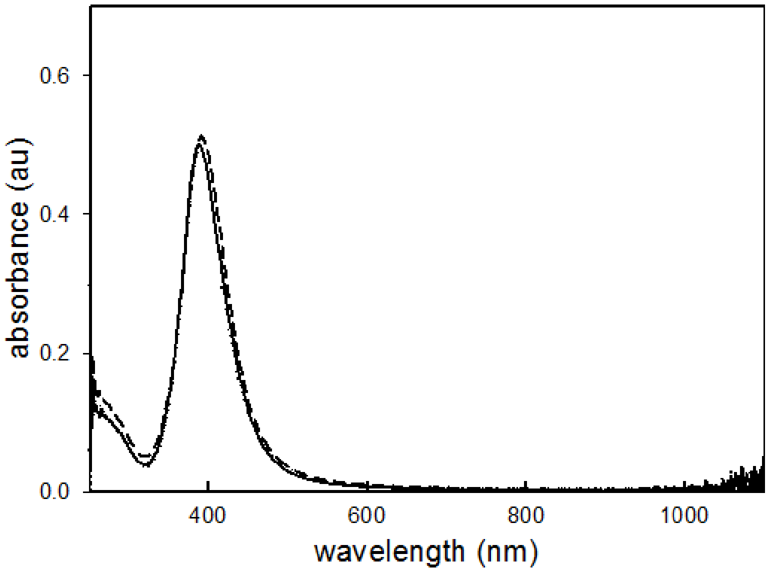

Figure S2. UV-vis spectra of three different stock solutions of seeds, after dilution to obtain an LSPR band having an absorbance of 0.5.

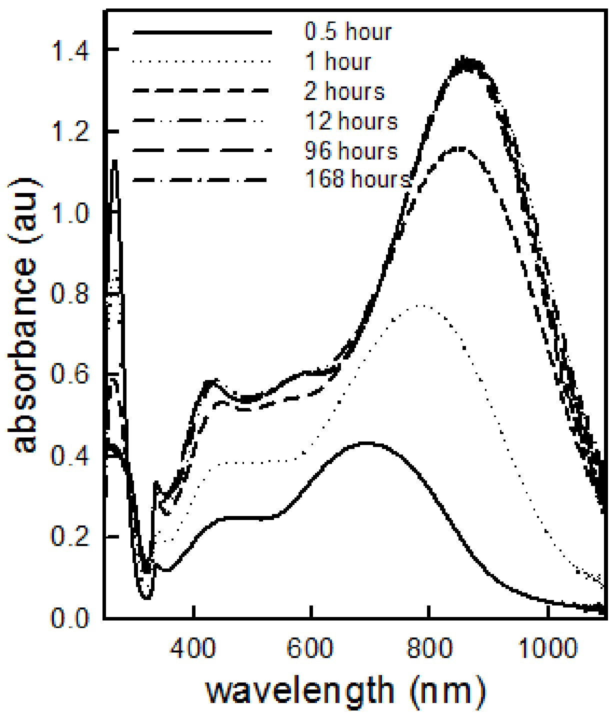

Figure S3. UV-vis spectra showing the kinetic of evolution with time of the seed-growth preparation of silver nanoplates, with *t* = 0 being the seed solution introduction into the growth solution.

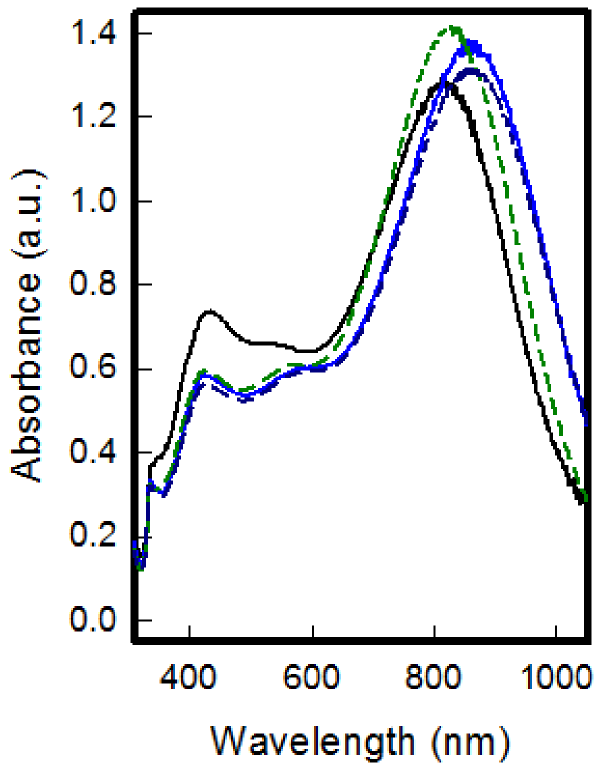

Figure S4. UV-vis spectra of four colloidal supensions of silver nanoplates obtained with the standard preparation.

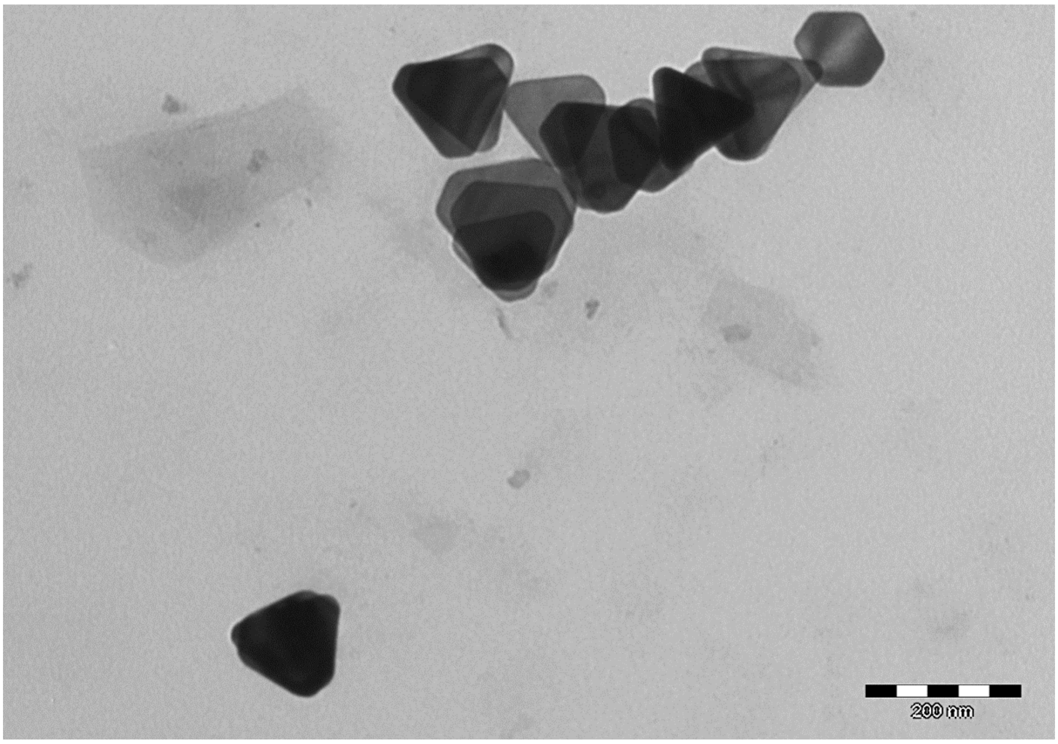

Figure S5. TEM image for a standard nanoplates preparation, showing the presence of snipped triangular and hexagonal objects, together with regular triangular objects.

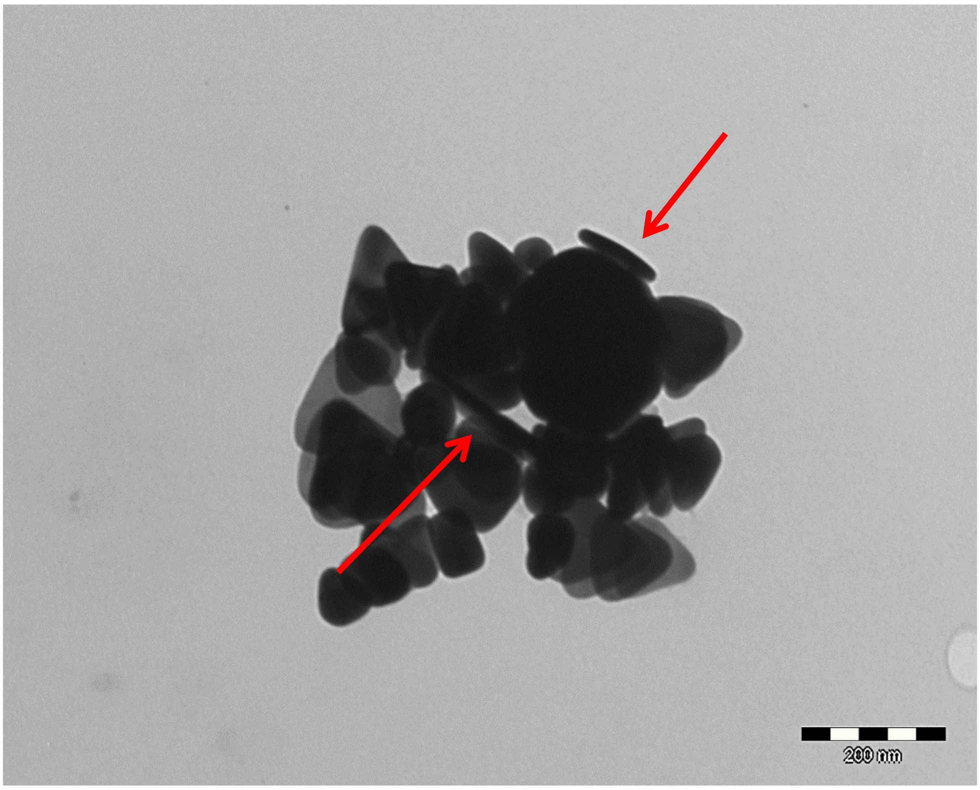

Figure S6. TEM image for a standard nanoplates preparation, showing the presence of a majority of triangular objects, with objects of various dimensions morphology, and of vertically placed objects (see arrows) with a thickness of about 20 nm.

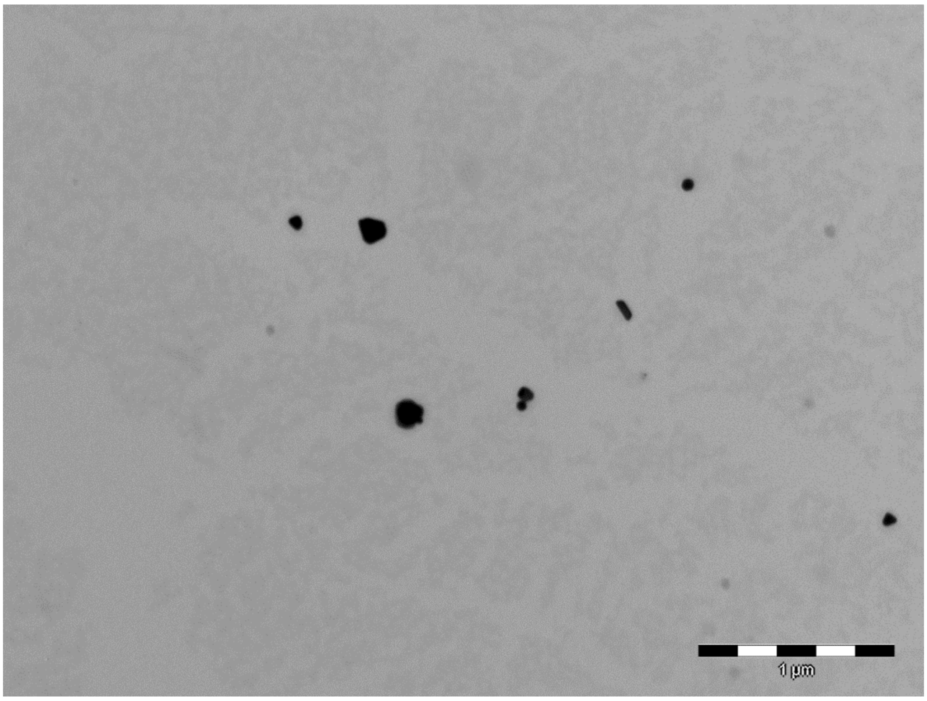

(**a**)

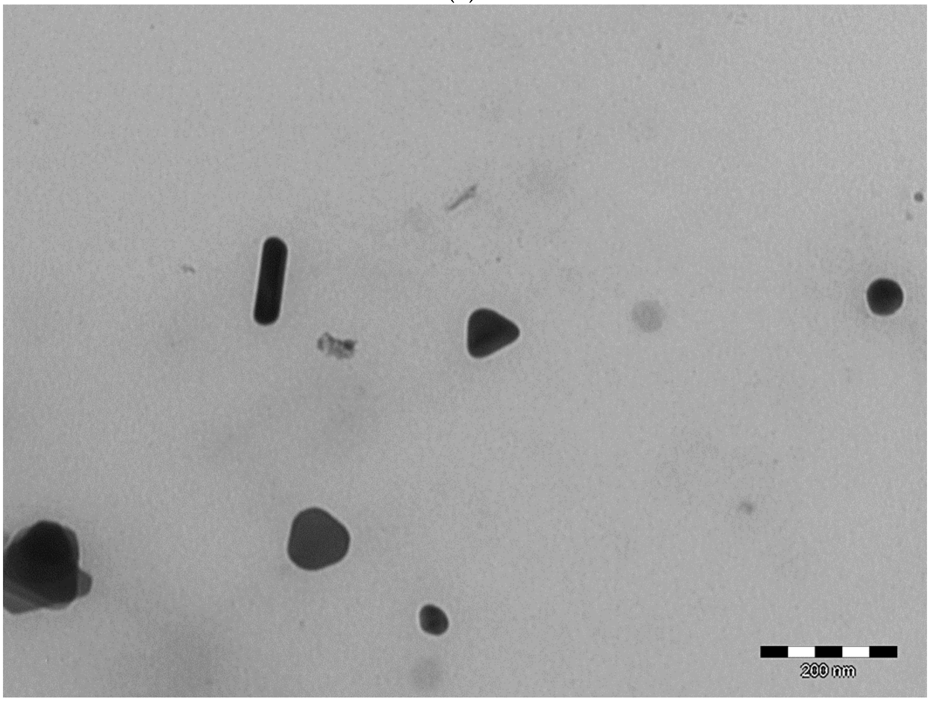

(**b**)

Figure S7. TEM images for a standard nanoplates preparation showing the existence of a few rod-like and rounded objects. (**a**) Scale 1 micron; (**b**) Scale 200 nm.

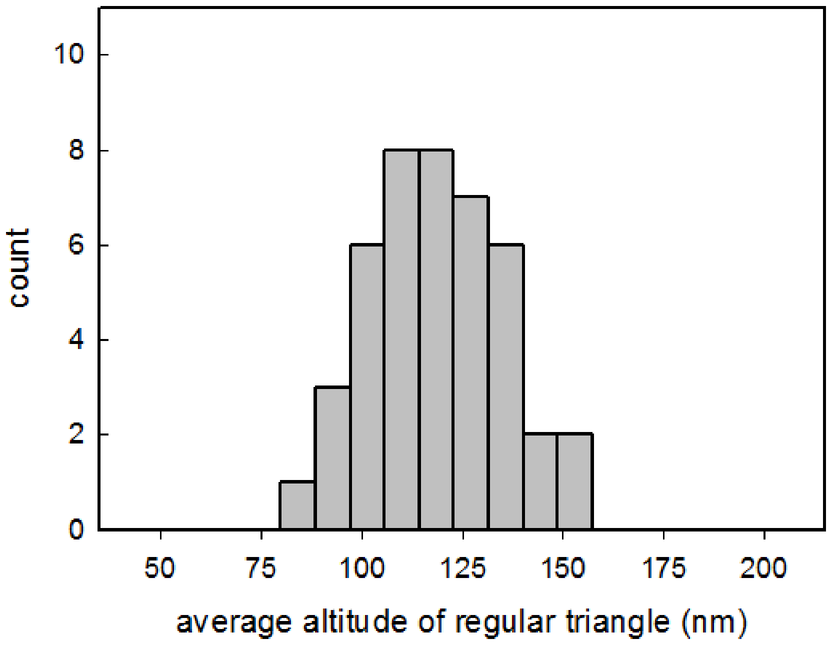

Figure S8. Histogram representing the distribution of the average altitude values measured for 43 regular triangles obtained from the standard preparation reported in Figure. 1a of the main text.

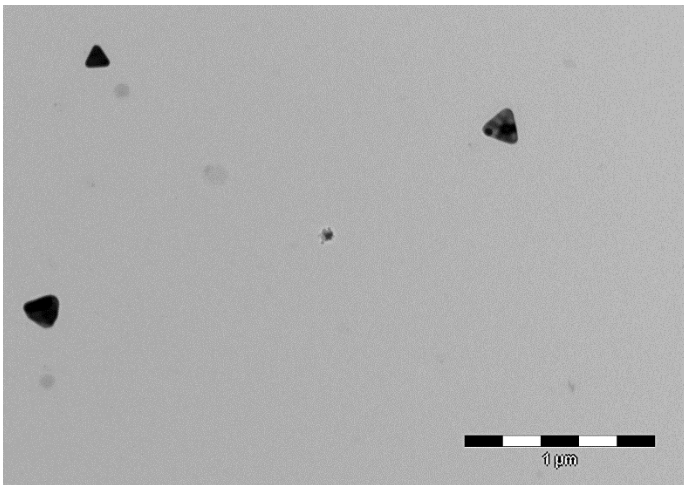

(**a**)

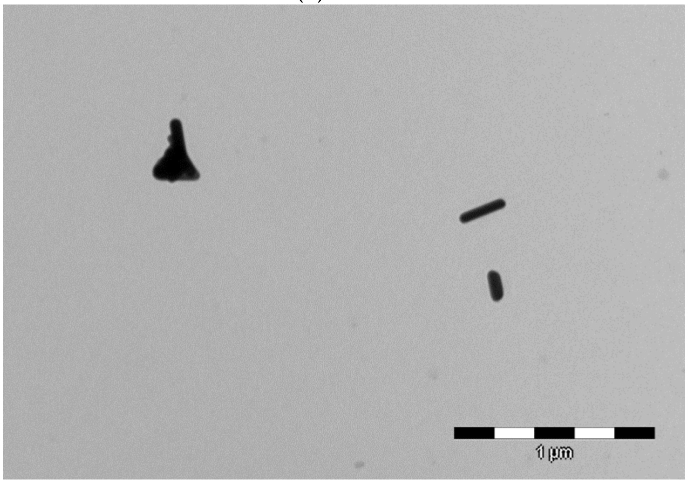

(**b**)

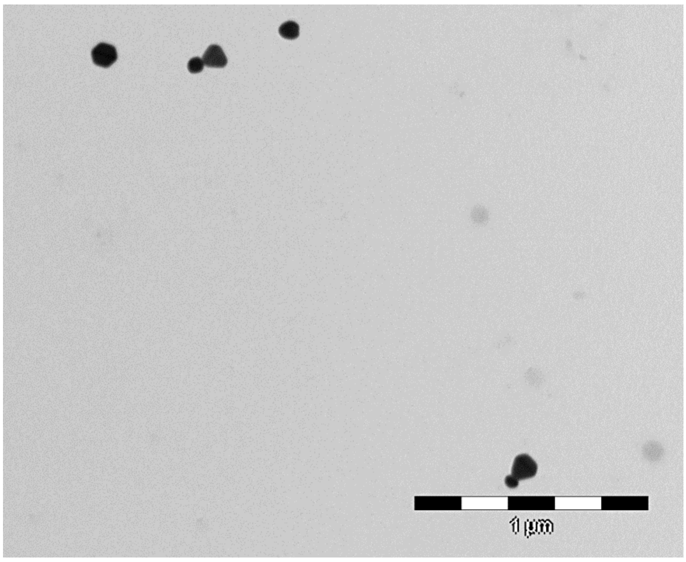

(**c**)

Figure S9. (**a**–**c**) TEM images of nanoplates obtained decreasing of one third the seed quantity (in respect to the standard preparation). Scale bar: 1 micron.

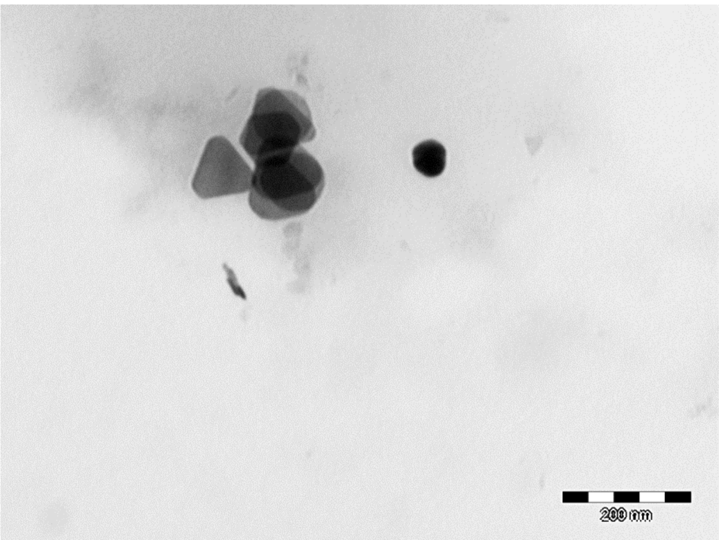

(**a**)

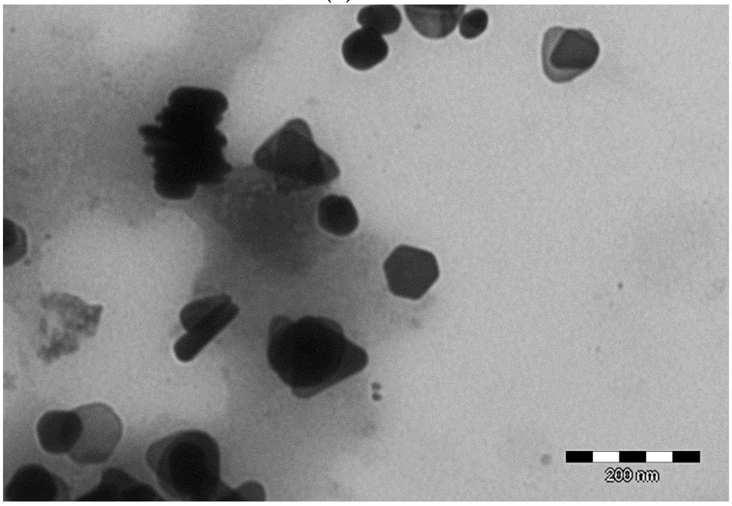

(**b**)

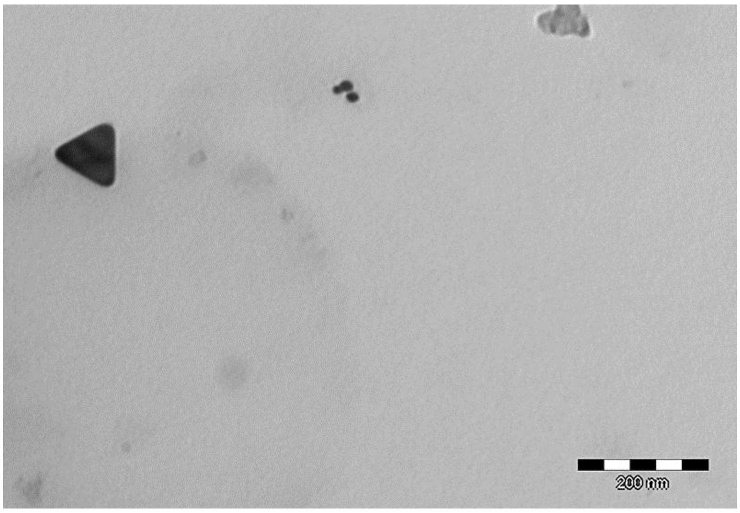

(**c**)

Figure S10. (**a**–**c**) TEM images of nanoplates obtained doubling the seed quantity (in respect to the standard preparation). Scale bar: 200 nm.

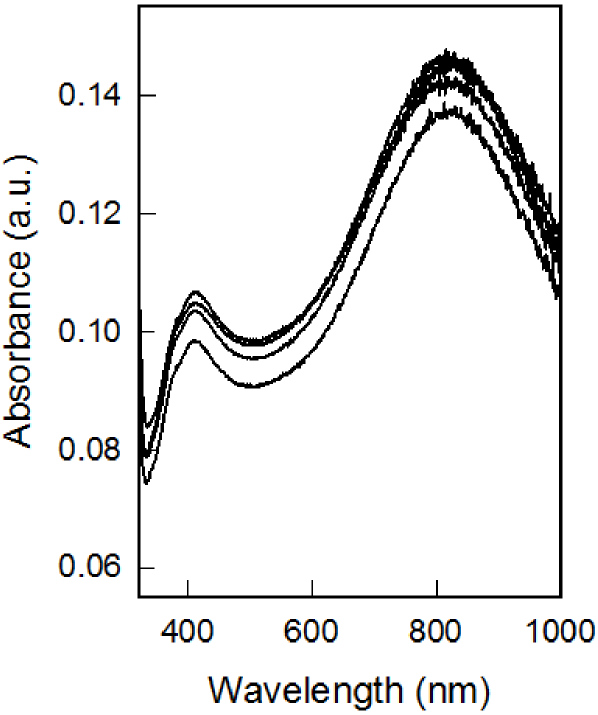

Figure S11. UV-vis spectra of four GLASS-PEI-TRI samples, obtained from grafting of nanoplates on PEI functionalized glass samples (GLASS-PEI), coming from four identical preparations performed in four diffferent reaction vessels.

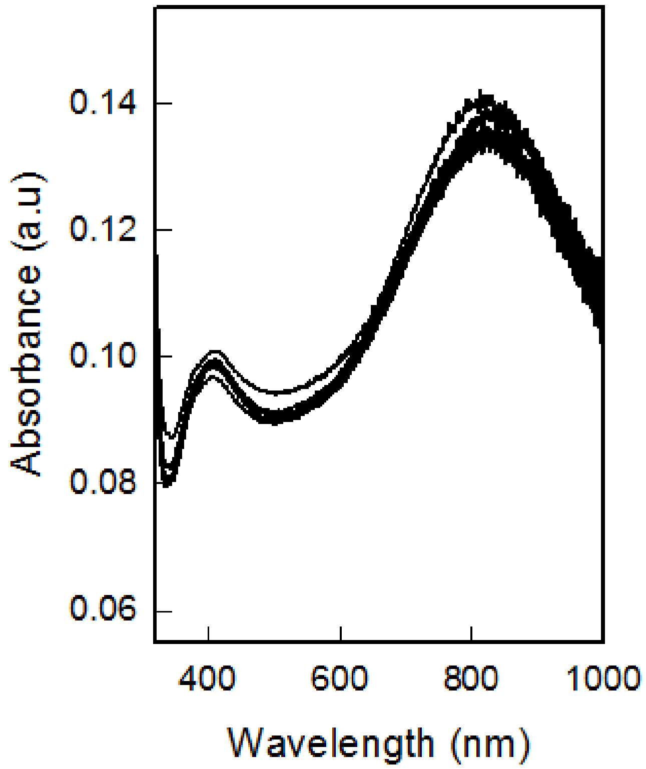

Figure S12. UV-vis spectra of seven GLASS-PEI-TRI samples obtained in the same reaction vessel during a single preparation.

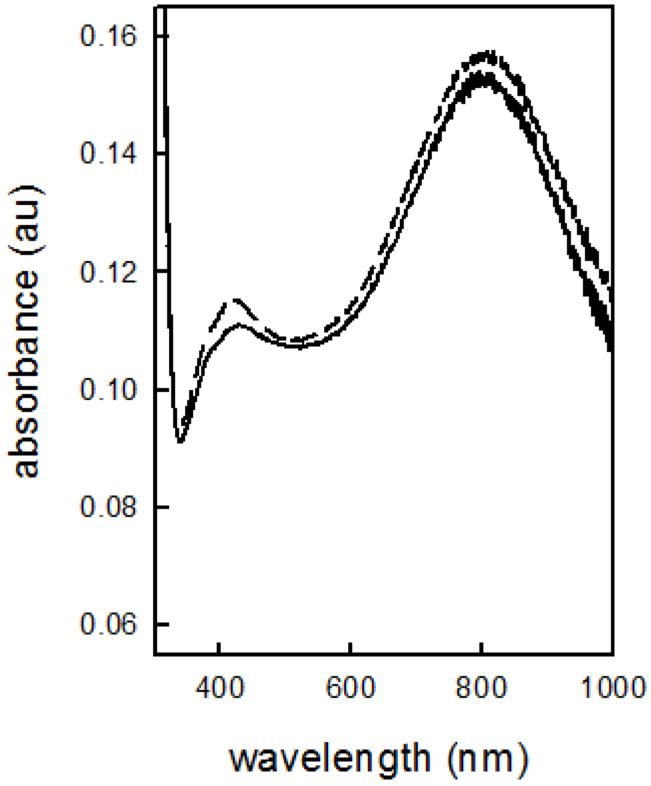

Figure S13. UV-vis spectra of the same GLASS-PEI-TRI sample before (solid line) and after (dashed line) immersion in water for 48 hours.

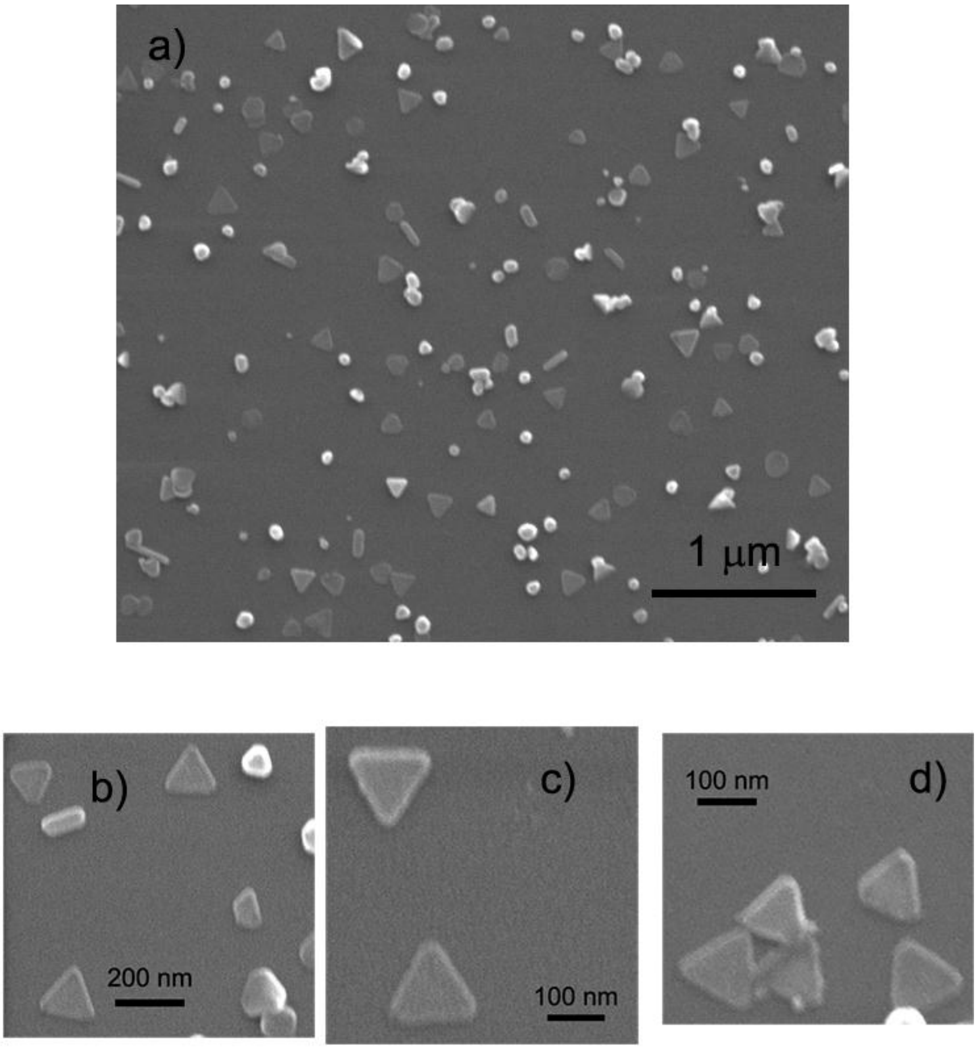

Figure S14. SEM images of a sample of GLASS-PEI-TRI after 48 h of immersion in water: (**a**) large area image; (**b–d**) images of triangular objects, which retains morphology and dimension observed in the fresh samples.

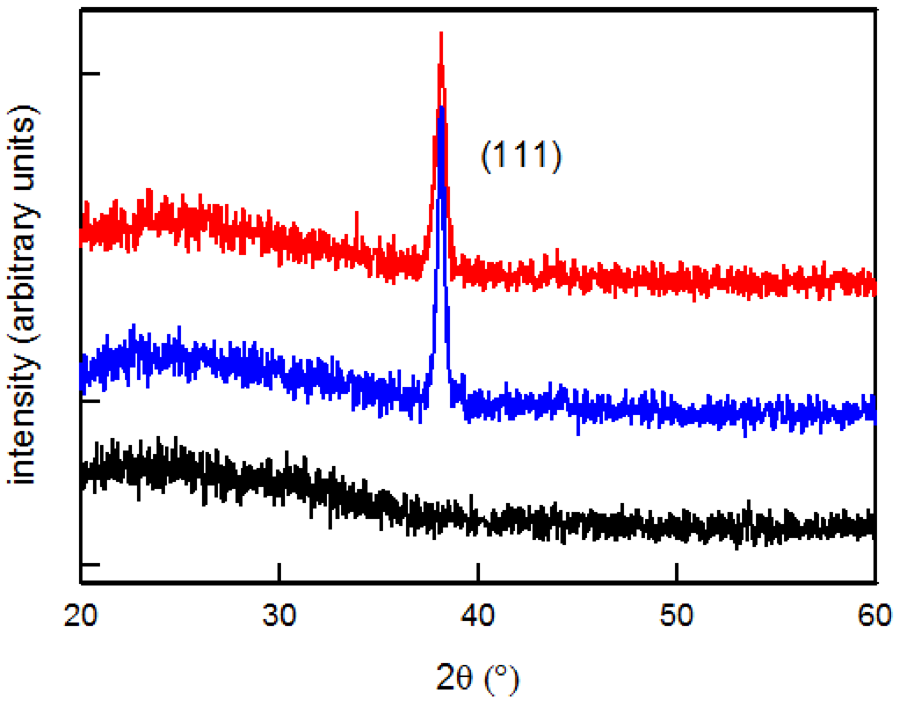

Figure S15. XRD patterns obtained for a blank glass (black line), for a sample of nanoplates centrifuged, concentrated, placed on a blank glass and dried as a powder (blue line), and for a GLASS-PEI-TRI sample (red line), evidencing the presence of (111) diffraction peak.

© 2017 by the authors. Submittedfor possible open access publication under the terms and conditions of the Creative Commons Attribution (CC-BY) license (http://creativecommons.org/licenses/by/4.0/).
